# 3D *in vitro* Model of Vascular Medial Thickening in Pulmonary Arterial Hypertension

**DOI:** 10.3389/fbioe.2020.00482

**Published:** 2020-05-20

**Authors:** Chiharu Morii, Hiroyoshi Y. Tanaka, Yasuhisa Izushi, Natsumi Nakao, Masaya Yamamoto, Hiromi Matsubara, Mitsunobu R. Kano, Aiko Ogawa

**Affiliations:** ^1^Department of Pharmaceutical Biomedicine, Graduate School of Medicine, Dentistry, and Pharmaceutical Sciences, Okayama University, Okayama, Japan; ^2^Division of Molecular and Cellular Medicine, Department of Clinical Science, National Hospital Organization Okayama Medical Center, Okayama, Japan; ^3^Department of Materials Processing, Graduate School of Engineering, Tohoku University, Sendai, Japan; ^4^Graduate School of Biomedical Engineering, Tohoku University, Sendai, Japan; ^5^Department of Pharmaceutical Biomedicine, Graduate School of Interdisciplinary Science and Engineering in Health Systems, Okayama University, Okayama, Japan

**Keywords:** pulmonary arterial hypertension, medial thickening, pulmonary artery smooth muscle cell, 3D culture, PDGF signaling, imatinib

## Abstract

In pulmonary arterial hypertension (PAH), excessive proliferation of pulmonary artery smooth muscle cells (PASMCs) causes vascular medial thickening. Medial thickening is a histopathological hallmark of pulmonary vascular remodeling, the central disease process driving PAH progression. Pulmonary vascular remodeling causes stenosis and/or obstruction of small pulmonary arteries. This leads to increased pulmonary vascular resistance, elevated pulmonary arterial pressure, and ultimately right heart failure. To improve the survival of PAH patients, which remains at approximately 60% at 3 years after diagnosis, the development of novel PAH-targeted drugs is desired. To this end, a detailed understanding of the mechanisms underlying excessive PASMC proliferation and the medial thickening that ensues is necessary. However, a lack of *in vitro* models that recapitulate medial thickening impedes our deeper understanding of the pathogenetic mechanisms involved. In the present study, we applied 3-dimensional (3D) cell culture technology to develop a novel *in vitro* model of the pulmonary artery medial layer using human PAH patient-derived PASMCs. The addition of platelet-derived growth factor (PDGF)-BB, a mitogen known to promote excessive PASMC proliferation in PAH, resulted in increased thickness of the 3D-PAH media tissues. Conversely, administration of the PDGF receptor inhibitor imatinib or other clinical PAH drugs inhibited this medial thickening-inducing effect of PDGF-BB. Altogether, by using 3D cell culture technology, we report the generation of an *in vitro* model of medial thickening in PAH, which had hitherto not been successfully modeled *in vitro*. This model is potentially useful for assessing the ability of candidate PAH drugs to suppress medial thickening.

## Introduction

Pulmonary arterial hypertension (PAH) is a devastating disease. In PAH, pulmonary vascular remodeling causes stenosis and/or obstruction of small pulmonary arteries. This leads to increased pulmonary vascular resistance, elevated pulmonary arterial pressure, and ultimately right heart failure. The survival rate is approximately 60% at 3 years after diagnosis despite decades of progress in pulmonary artery-specific vasodilators targeting the three main therapeutic pathways (i.e., bosentan, ambrisentan and macitentan targeting the endothelin pathway; selexipag, beraprost, iloprost, treprostinil, and epoprostenol targeting the prostacyclin pathway; tadalafil, vardenafil, and sildenafil targeting the nitric oxide pathway) (Humbert et al., 2010; Benza et al., 2012; Galiè et al., 2019). There is thus a demand for the development of novel PAH-targeted drugs (Antoniu, 2012; Hemnes and Humbert, 2017).

Excessive proliferation of pulmonary artery smooth muscle cells (PASMCs) is the main driver of pulmonary vascular remodeling (Hassoun et al., 2009; Morrell et al., 2009). Decreased apoptosis of PASMCs has also been noted, which further results in increased number of PASMCs (Gurbanov and Shiliang, 2006). It is thus of paramount importance to elucidate the mechanisms driving excessive PASMC proliferation and to gain a better understanding of the pathobiology of PAH. Clinically relevant models of PAH are necessary to achieve this task. There are several experimental animal models to explore the mechanisms by which PAH develops. Classical animal models of PAH were based either on chronic hypoxia or monocrotaline injury (Stenmark et al., 2009; Gomez-Arroyo et al., 2011; Maarman et al., 2014). However, certain histopathological features of human PAH were not recapitulated faithfully in these animal models, such as irreversible intimal fibrosis or plexogenic arteriopathy which result in the obliteration of the small/medium-sized pulmonary arteries (Stenmark et al., 2009; Carman et al., 2019). Furthermore, it has been suggested that with more than 30 agents being reported to prevent and/or reverse the monocrotaline-induced pulmonary hypertension (paradoxically even including those that have been implicated in the development of PAH), the model is relatively easy to therapeutically improve—unlike human PAH (Stenmark et al., 2009). Numerous animal models beyond the chronic hypoxia and monocrotaline injury models are being developed, and it is becoming clear that models involving multiple pathological insults (such as the addition of SU5416 to the chronic hypoxia model to exacerbate endothelial injury) better mimic severe PAH in humans (Maarman et al., 2014; Carman et al., 2019).

In addition to animal models of PAH, the use of patient-derived PASMCs offers a unique opportunity to understand the pathogenetic mechanisms at play in PAH and to investigate the effectiveness of novel drug candidates. Indeed, we and others have reported disease-specific phenotypes of PASMCs derived from patients with PAH, especially in regards to platelet-derived growth factor (PDGF) signaling (Ogawa et al., 2005, 2008; Perros et al., 2008; Nakamura et al., 2012). Five types of PDGF ligands (PDGF-AA, PDGF-AB, PDGF-BB, PDGF-CC, and PDGF-DD) result from the dimerization of the four PDGF isoforms (PDGF-A, PDGF-B, PDGF-C, and PDGF-D), and signal through three types of PDGF receptors (PDGFR-αα, PDGFR-αβ, and PDGFR-ββ) that result from the dimerization of the two receptor isoforms (PDGFR-α and PDGFR-β) (Andrae et al., 2008). Notably, the expression levels of PDGF-A, PDGF-B, and both PDGF receptor isoforms have been reported to be elevated in PAH (Schermuly et al., 2005; Perros et al., 2008). Indeed, PAH patient-derived PASMCs demonstrate an increased proliferative response to PDGF-BB that is blocked by imatinib (Ogawa et al., 2005, 2008; Perros et al., 2008; Nakamura et al., 2012). Imatinib is a small molecular weight receptor tyrosine kinase inhibitor clinically approved for use against chronic myeloid leukemia and gastrointestinal tumors, with inhibitory activity against both isoforms of PDGF receptors (Cohen et al., 2002; Lopes and Bacchi, 2010). The up-regulation of PDGF signaling components, the enhanced proliferative response to PDGF signaling, and the ability of imatinib to reverse experimental PAH led to imatinib being considered a candidate PAH drug (Ogawa et al., 2005; Schermuly et al., 2005; Perros et al., 2008; Ghofrani et al., 2010; Nakamura et al., 2012; Hoeper et al., 2013).

Notably, however, most published cell studies were performed in 2-dimensional (2D) cell culture systems (i.e., cellular monolayers cultured in plastic dishes) mainly for their technical ease. However, 2D cell culture fails to recapitulate important features of the microenvironment in which the cells are situated *in vivo* (Baker and Chen, 2012). For example, 2D cell culture cannot mimic vascular medial thickening, which is a histopathological hallmark of PAH (Stenmark et al., 2018). Given that the ultimate goal of PAH therapy is to halt and/or reverse vascular remodeling and the medial thickening that accompanies, a 3-dimensional (3D) model of vascular medial thickening in PAH would be useful for the discovery and assessment of novel drug candidates. There are, however, no reports to date of a 3D model consisting of PAH patient-derived PASMCs.

We report the construction of a novel 3D model of the vascular media layer in PAH using PAH patient-derived PASMCs. PDGF signaling increased the thickness of the 3D-PAH media tissues while treatment with imatinib, an inhibitor of PDGF receptors and a candidate PAH drug, decreased thickness. Importantly, change in the thickness of the 3D-PAH media tissue generally correlated with changes in the expression of proliferation markers. Furthermore, imatinib induced apoptosis of PASMCs within the 3D-PAH media tissues. Finally, we tested clinical PAH drugs for its effect on the thickness of 3D-PAH media tissues. Altogether, we report a novel *in vitro* model of medial thickening in PAH with potential applications in drug testing.

## Materials and Methods

### Histological Staining of Human Lung Tissue

Lung tissue was obtained from a patient with PAH by autopsy at National Hospital Organization Okayama Medical Center (Okayama, Japan) after written informed consent was obtained from the next of kin. Elastica Masson staining was performed on formalin-fixed, paraffin-embedded sections.

### Cell Culture and Reagents

Pulmonary artery smooth muscle cells were isolated from the lungs of 3 patients with PAH who either underwent autopsy (patient #1 and #2) or lung transplantation (patient #3). All experiments were carried out after approval by the Institutional Review Board of National Hospital Organization Okayama Medical Center. Written informed consent was obtained from either the patient or the next of kin before the procedure. Cell isolation was performed as previously reported (Ogawa et al., 2005; Fujio et al., 2006). PASMCs were cultured on Collagen I-coated dishes (IWAKI/AGC TECHNO GLASS Co., Ltd., Shizuoka, Japan), in Dulbecco’s Modified Eagle Medium [low glucose (1 g/L); gibco/Thermo Fisher Scientific, Waltham, MA, United States] containing 10% (v/v) fetal bovine serum with 1% (v/v) Penicillin-Streptomycin (gibco/Thermo Fisher Scientific). Cells were incubated at 37°C in a humidified 5% CO_2_ atmosphere. After reaching confluence, the cells were sub-cultured by trypsinization with TrypLE Express (gibco/Thermo Fisher Scientific) at sub-cultivation ratios between 1:3 and 1:4 with a time to confluence of between 3 and 5 days across PASMCs.

### Generation of 3D-PAH Media Tissues

3-dimensional PAH media tissues were generated using a 3D cell culture technique reported previously (Tanaka et al., 2019). Briefly, trypsinized PASMCs were first incubated in Tris–buffered saline (pH 7.4) containing 0.04 mg/mL Fibronectin (Sigma-Aldrich, St. Louis, MO, United States) and 0.04 mg/mL Gelatin (Wako Pure Chemicals, Osaka, Japan) upon gentle rocking [30 min, room temperature (RT)]. 5.0 × 10^5^ PASMCs were then seeded on cell culture inserts for 24 well plates (0.4 μm, transparent; BD Falcon/Corning, Corning, NY, United States) coated with 0.12 mg/mL Fibronectin. After 3 days of incubation, 3D-PAH media tissues were fixed with 4% (w/v) paraformaldehyde (PFA) in phosphate-buffered saline (PBS; 20 min, RT) for hematoxylin-eosin staining (Applied Medical Research, Osaka, Japan) and observed under a BioRevo BZ-9000 fluorescence microscope (Keyence, Osaka, Japan).

### Evaluation of 3D-PAH Media Tissue Thickness

To assess the effect of PDGF-BB on 3D-PAH media tissue thickness, 3D-PAH media tissues were treated with or without 10 ng/mL PDGF-BB (Sigma-Aldrich) from 4 h post cell seeding. To test the effect of various inhibitors on 3D-PAH media tissue thickness, inhibitors were administered in addition to PDGF-BB. The inhibitors used in this study were imatinib [1 μg/mL in dimethyl sulfoxide (DMSO); Selleck Chemicals, Houston, TX, United States], bosentan (50 μM in DMSO; USP, Rockville, MD, United States), MRE-269 (400 nM in DMSO; Cayman Chemicals, Ann Arbor, MI, United States), and tadalafil (200 nM in DMSO; Sigma-Aldrich).

In all experiments, 3D-PAH media tissues were collected 72 h post cell seeding. 3D-PAH media tissues were washed once with PBS, fixed with 4% (w/v) PFA in PBS (5 min, RT) and permeabilized with 0.2% (v/v) Triton X-100 in PBS (20 min, RT). Nuclei were then stained with SYTOX Green nucleic acid stain (0.2 μM, 15 min, RT; Molecular Probes/Thermo Fisher Scientific, Eugene, OR, United States). After washing with PBS thrice, culture insert membranes were carefully excised using a scalpel and mounted on coverslips using fluorescent mounting medium (Dako/Agilent, Santa Clara, CA, United States). Samples were then observed under a Nikon C2+ confocal laser microscope (Tokyo, Japan), and Z-stack images of 0.1 μm slices were obtained. Images were 3D-reconstituted and the thickness determined using the NIS-Elements AR version 4.30 software (Nikon).

### Immunofluorescent Staining of 3D-PAH Media Tissues

3-dimensional PAH media tissues were washed once with PBS, fixed with 4% (w/v) PFA in PBS (10 min, RT), permeabilized with 0.2% (v/v) Triton X-100 in PBS (5 min, RT), and blocked with Blocking One (nacalai tesque, Kyoto, Japan; >2 h, RT). The 3D-PAH media tissues were then incubated overnight at 4°C with Anti-Ki67 antibody (ab15580, rabbit polyclonal, Abcam, Cambridge, United Kingdom; final 1 μg/mL) diluted in Blocking One. After washing with PBS, 3D-PAH media tissues were incubated with Alexa Fluor 594-labeled donkey anti-rabbit IgG (H + L) secondary antibody (A-21207, Molecular Probes/Thermo Fisher Scientific; dilution: 1/200) diluted in Blocking One (30 min, RT). Finally, nuclei were stained with SYTOX Green (0.2 μM, 15 min, RT). Samples were observed under a Nikon C2+ confocal laser microscope. To quantify Ki67-positive cell nuclei, the area of Ki67-positive nuclei was divided by total nuclei area for four randomly chosen visual fields using ImageJ (National Institute of Health, Bethesda, MD, United States).

### Terminal Deoxynucleotidyl Transferase dUTP Nick end Labeling (TUNEL) Staining of 3D-PAH Media Tissues

Apoptotic PASMCs were detected using the *in situ* Cell Death Detection Kit, TMR-red (Roche, Basel, Switzerland) according to the manufacturer’s protocol. Briefly, 3D-PAH media tissues were washed once with PBS, fixed with 4% (w/v) PFA in PBS (10 min, RT), and permeabilized with 0.2% (v/v) Triton X-100 in PBS (1 h, 37°C). After washing, the labeling reaction was performed with the addition of the TUNEL reaction mixture (24 h, 4°C). After washing with PBS, nuclei were stained with SYTOX Green (0.2 μM, 15 min, RT). Samples were observed under a Nikon C2+ confocal laser microscope. For quantification, the area of TUNEL-positive nuclei was divided by total nuclei area for four randomly chosen visual fields using ImageJ.

### Reverse Transcription-Quantitative Polymerase Chain Reaction (RT-qPCR)

Seventy two hours post cell seeding, total RNA was isolated from 3D-PAH media tissues using TRI Reagent (Molecular Research Center, Cincinnati, OH, United States) and reverse transcribed to complementary DNA (ReverTra Ace -α-; TOYOBO, Osaka, Japan) according to the manufacturer’s protocol. RT-qPCR was performed using the THUNDERBIRD SYBR qPCR mix (TOYOBO) on the StepOne Plus real-time PCR system (Applied Biosystems, Foster City, CA, United States). Primers (Fasmac Co., Kanagawa, Japan) used in this study were: *CCND1* (which encodes Cyclin D1; Forward: 5′-AATGAAGCCAGCTCACAGTG-3′, Reverse: 5′-GCGGGGTGCAAATTCTTTTG-3′) and *ACTB* (which encodes β-actin; Forward: 5′-TCACCCACACTGTGCCCATCTACGA-3′, Reverse: 5′-CAGCGGAACCGCTCATTGCCAATGG-3′). *ACTB* was used as the internal control for all RT-qPCR experiments.

### Statistical Analysis

All data are expressed as mean ± standard deviation. Sample sizes are indicated in the respective figure legends or individual data points directly shown in the graph. Statistical analyses were performed using GraphPad Prism 8 (GraphPad Software, Inc., La Jolla, CA, United States). For experiments with two experimental groups, unpaired Student’s *t*-test with Welch’s correction was performed. For experiments with more than three experimental groups, one-way analysis of variance followed by *post hoc* Dunnett’s multiple comparisons test was performed. For all analyses, statistical significance was set at *p* < 0.05. In all figures: *n.s.*, ^∗^, ^∗∗^, ^∗∗∗^, and ^****^ denote not significant, *p* < 0.05, *p* < 0.01, *p* < 0.001, *p* < 0.0001, respectively.

## Results

### Generation of 3D-PAH Media Tissues

**FIGURE 1:**
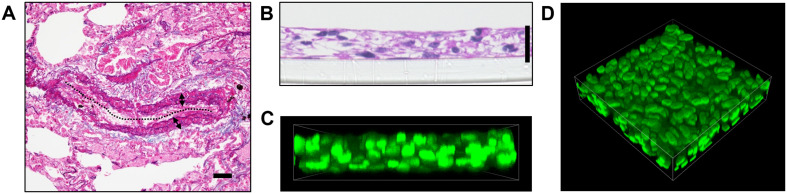
Generation of 3D-PAH media tissues. **(A)** Representative elastic tissue staining of a pulmonary artery in a patient with pulmonary arterial hypertension (PAH). The dotted line indicates vascular lumen, double-headed arrows the medial layer. Scale bar = 50 μm. **(B)** Representative hematoxylin-eosin staining of 3-dimensional (3D) model of the pulmonary artery vascular media in PAH (hereafter, 3D-PAH media tissues). Scale bar = 20 μm. **(C,D)** Representative side view **(C)** and 3D view **(D)** of 3D-PAH media tissues with the nuclei of pulmonary arterial smooth muscle cells (PASMCs) stained (SYTOX Green; green). PASMCs derived from PAH patient #1 was used for construction of 3D-PAH tissues shown in **(B–D)**.

As a result of pulmonary vascular remodeling, pulmonary arteries in patients afflicted with PAH demonstrate prominent medial thickening which consists of multiple layers of PASMCs (Figure 1A). We thus sought to recapitulate this *in vitro* by using a 3D cell culture technique that we have previously reported (Tanaka et al., 2019). By seeding 5 × 10^5^ human PAH patient-derived PASMCs into 24 well cell culture inserts, we obtained a 3D structure consisting of 4–6 layers of PASMCs with a thickness of between 20 and 30 μm (Figures 1B–D).

### PDGF-BB Induces Thickening of 3D-PAH Media Tissues

**FIGURE 2:**
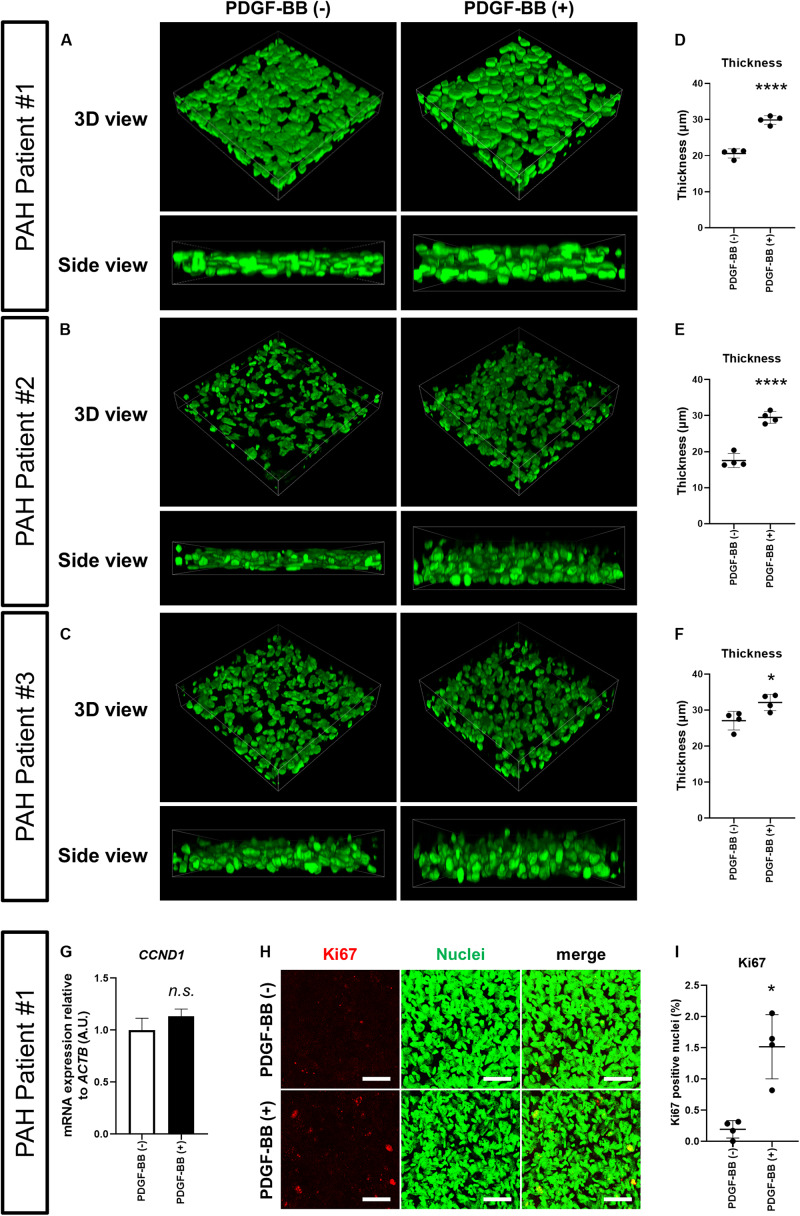
PDGF-BB induces thickening of 3D-PAH media tissues. **(A–C)** Representative 3D-reconstructed images of 3D-PAH media tissues cultured with platelet-derived growth factor (PDGF)-BB [PDGF-BB (+)] or without PDGF-BB [PDGF-BB (-)]. 3D-PAH tissues were generated using PASMCs from three different PAH patients #1, #2, and #3. Side views are shown to facilitate the comparison of thickness. **(D–F)** Quantification of the thickness of 3D-PAH media tissues as shown in **(A–C)**, respectively. **(G)** Reverse transcription-quantitative polymerase chain reaction (RT-qPCR) analysis of *CCND1* expression in 3D-PAH media tissues cultured with or without PDGF-BB. *n* = 3 independently constructed 3D-PAH media tissues from PAH patient #1. **(H)** Representative images of 3D-PAH media tissues generated using PASMCs derived from PAH patient #1, cultured with or without PDGF-BB. 3D-PAH media tissues were stained for Ki67 (red). Nuclei were stained by SYTOX Green (green). Scale bars = 50 μm. **(I)** Quantification of Ki67-positive nuclear area as a percentage of the total nuclear area as shown in **(H)**. In **(D–G)** and **(I)**, *n.s.*, not significant, *p < 0.05, and ****p < 0.0001, unpaired Student’s *t*-test with Welch’s correction.

Platelet-derived growth factor-BB is a potent PASMC mitogen and elevated in the sera of PAH patients (Selimovic et al., 2009). Together with the finding that the expression of PDGF receptors in PAH patient-derived PASMCs is elevated (Perros et al., 2008), there is accumulating evidence that abnormal activation of PDGF signaling plays a critical role in PAH pathogenesis and progression (Schermuly et al., 2005; Perros et al., 2008; Grimminger and Schermuly, 2010; Antoniu, 2012; ten Freyhaus et al., 2012). We, therefore, wondered whether we could model the process of medial thickening by treating the 3D-PAH media tissues with PDGF-BB. Indeed, the thickness of 3D-PAH media tissues treated with PDGF-BB was greater than tissues without PDGF-BB treatment (Figures 2A–F). We then asked whether this increase in thickness might be due to the increased proliferation of PASMCs induced by PDGF signaling. While we did not observe a change in the mRNA expression of the proliferation marker Cyclin D1 (encoded by *CCND1* gene; Figure 2G) upon treatment of 3D-PAH media tissues with PDGF-BB, an increase in the proportion of nuclei positive for the proliferation marker Ki67 was observed (Figures 2H,I).

### Imatinib Inhibits Thickening of 3D-PAH Media Tissues Induced by PDGF-BB via Suppressing Proliferation and Inducing Apoptosis of PASMCs

**FIGURE 3:**
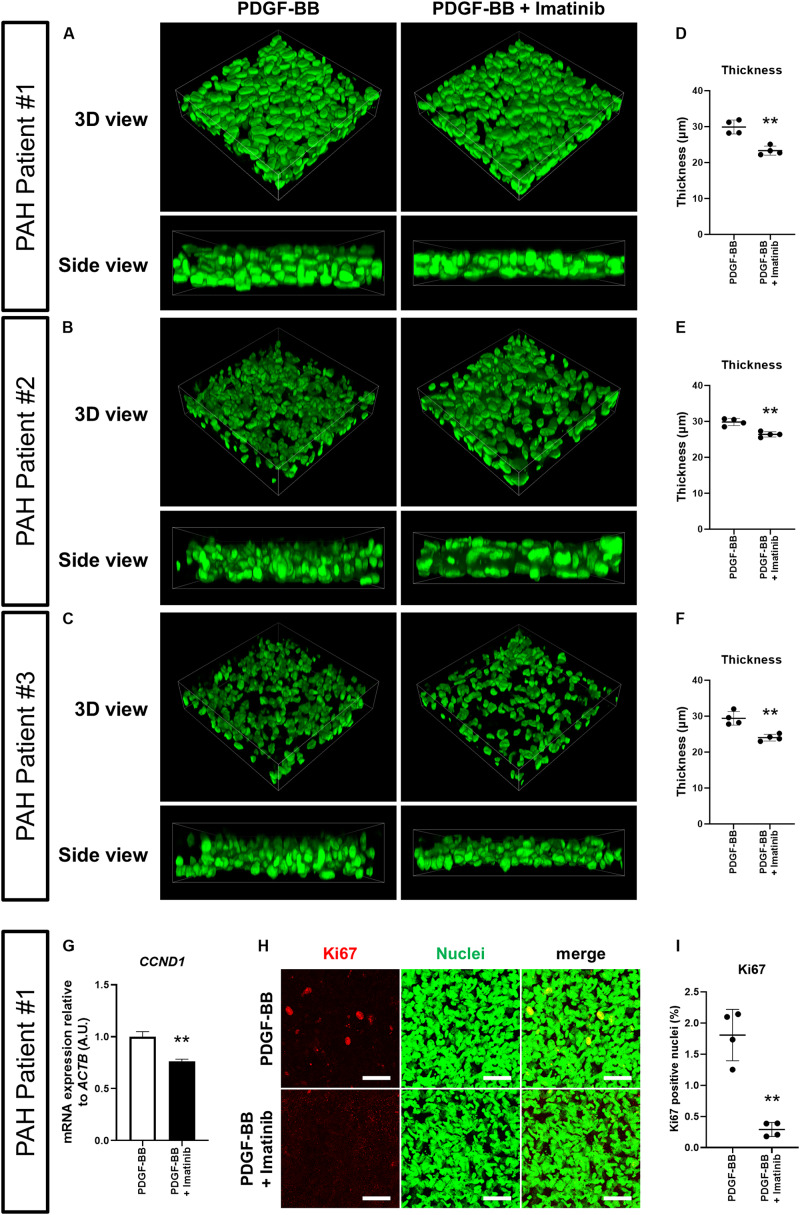
Imatinib inhibits the thickening of 3D-PAH media tissues induced by PDGF-BB via suppressing proliferation of PASMCs. **(A–C)** Representative 3D-reconstructed images of 3D-PAH media tissues cultured in the presence of PDGF-BB with or without imatinib treatment. 3D-PAH tissues were generated using PASMCs from three different PAH patients #1, #2, and #3. Side views are shown to facilitate the comparison of thickness. **(D–F)** Quantification of the thickness of 3D-PAH media tissues as shown in **(A–C)**, respectively. **(G)** RT-qPCR analysis of *CCND1* expression in 3D-PAH media tissues cultured in the presence of PDGF-BB with or without imatinib treatment. *n* = 3 independently constructed 3D-PAH media tissues from PAH patient #1. **(H)** Representative images of 3D-PAH media tissues generated using PASMCs derived from PAH patient #1, cultured in the presence of PDGF-BB with or without imatinib treatment. 3D-PAH media tissues were stained for Ki67 (red). Nuclei were stained by SYTOX Green (green). Scale bars = 50 μm. **(I)** Quantification of Ki67-positive nuclear area as a percentage of the total nuclear area as shown in **(H)**. In **(D–G)**, and **(I)**, ^∗∗^*p* < 0.01, unpaired Student’s *t*-test with Welch’s correction.

To assess whether our 3D-PAH medial thickening model could be used for drug testing, we first asked whether we could inhibit the PDGF-BB-induced thickening of 3D-PAH media tissues by administering imatinib, a pharmacological inhibitor of PDGF signaling. Indeed, treatment with imatinib reduced the thickness of 3D-PAH media tissues cultured in the presence of PDGF-BB (Figures 3A–F). In line with these findings, treatment with imatinib decreased *CCND1* mRNA expression (Figure 3G) and the proportion of Ki67-positive nuclei (Figures 3H,I). Furthermore, an increase in apoptotic PASMCs was observed in 3D-PAH media tissues treated with imatinib (Supplementary Figures S1A,B).

### Effect of Clinical PAH Drugs on the Thickness of 3D-PAH Media Tissues

**FIGURE 4:**
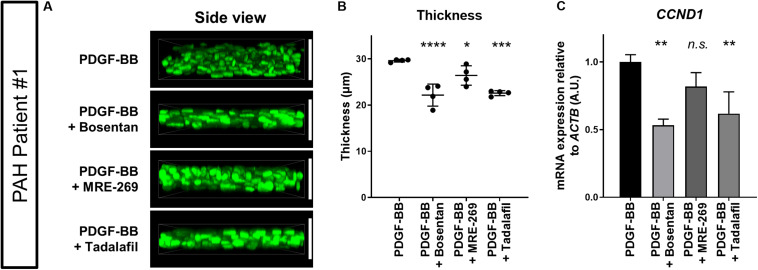
Effect of clinical PAH drugs on the thickness of 3D-PAH media tissues. **(A)** Representative 3D-reconstructed images of 3D-PAH media tissues cultured in the presence of PDGF-BB and treated with various clinical drugs. **(B)** Quantification of the thickness of 3D-PAH media tissues as shown in **(A)**. **(C)** RT-qPCR analysis of *CCND1* expression in 3D-PAH media tissues cultured in the presence of PDGF-BB and treated with various clinical PAH drugs. *n* = 3 independently constructed 3D-PAH media tissues from a single PAH patient. White vertical bars are shown to facilitate the comparison of thickness and indicate the thickness of 3D-PAH media tissue under the PDGF-BB condition. In **(B)** and **(C)** *n.s.*, not significant, *p < 0.05, **p < 0.01, ***p < 0.001, and ****p < 0.0001, one-way analysis of variance followed by *post hoc* Dunnett’s multiple comparisons test. All experiments shown in this figure were performed on PASMCs derived from PAH patient #1.

We finally asked whether clinical PAH drugs could inhibit the thickening of 3D-PAH media tissues induced by PDGF-BB. To this end, we chose to test bosentan, MRE-269 (the active metabolite of selexipag), and tadalafil. Each drug represents the three main therapeutic pathways in PAH: bosentan is an endothelin receptor antagonist that targets the endothelin pathway, MRE-269 is a prostacyclin receptor agonist that targets the prostacylcin pathway, and tadalafil is a phosphodiesterase 5 inhibitor that increases the intracellular concentration of cyclic GMP which is a signaling messenger molecule downstream of the nitric oxide signaling pathway (Humbert et al., 2004). We observed that all three drugs decreased the thickness of 3D-PAH media tissues cultured in the presence of PDGF-BB, with a more pronounced effect exerted by bosentan and tadalafil compared to MRE-269 (Figures 4A,B). Consistent with these findings, 3D-PAH media tissues treated with bosentan or tadalafil showed reduced *CCND1* mRNA expression even in the presence of PDGF-BB compared to 3D-PAH tissues cultured in the presence of PDGF-BB alone (Figure 4C). MRE-269 showed a tendency to decrease *CCND1* mRNA expression, but this trend did not reach statistical significance (Figure 4C). Furthermore, all three drugs decreased the proportion of Ki-67 positive nuclei (Supplementary Figures S2A,B) and increased the proportion of TUNEL-positive nuclei in 3D-PAH media tissues (Supplementary Figures S3A,B).

## Discussion

Pulmonary vascular remodeling affects all three layers of the pulmonary artery wall to varying extents, but medial involvement is almost always observed (Stenmark et al., 2018). The medial layer of the pulmonary artery in PAH patients is characteristically of greater thickness than its counterpart in healthy pulmonary arteries of similar caliber. Because of its central role in PAH pathobiology, medial thickening and the processes leading to it are deemed to be viable therapeutic targets in PAH. However, due to the essential lack of *in vitro* models of medial thickening, drug screening based on the ability of a compound to suppress medial thickening has hitherto been impossible. To the best of our knowledge, our 3D-PAH media model is the first application of 3D culture technology to model the vascular media using human PAH patient-derived PASMCs. Importantly, the modeling of the pulmonary artery medial layer in 3D enabled us to analyze the effect of various experimental conditions through assessing changes in the thickness of 3D-PAH media tissues.

Platelet-derived growth factor signaling has emerged, both clinically and experimentally, as an additional pathway of importance in PAH pathobiology apart from the three main pathways clinically targeted in PAH at present (Hemnes and Humbert, 2017). This can be ascribed to the pro-proliferative effect of PDGF signaling, as well as its role in inducing cell hypertrophy and promoting cell migration in vascular smooth muscle cells, all of which could contribute to vascular medial thickening (Yasunari et al., 1997; Perros et al., 2008). Indeed, using our 3D-PAH media model, we were able to recapitulate the process of medial thickening induced by PDGF-BB treatment. The increased thickness is at least partly due to increased PASMC proliferation induced by PDGF-BB treatment, although the contribution of PASMC hypertrophy and migration in our 3D-PAH media model warrants assessment in the future. Furthermore, imatinib treatment of the 3D-PAH media tissues resulted in decreased thickness and was associated with decreased proliferation and increased apoptosis of PASMCs. This is in line with experimental and initial clinical evidence showing the amelioration of PAH progression with imatinib treatment (Schermuly et al., 2005; Ghofrani et al., 2010; Nakamura et al., 2012; Hoeper et al., 2013). While the clinical implementation of imatinib in PAH treatment has so far proved difficult due to adverse events, side effects, and high rate of discontinuation (Frost et al., 2015), it remains plausible that other strategies to target the PDGF signaling pathway might prove viable. For example, efforts to identify downstream effectors of PDGF signaling involved in PAH progression have identified phosphatidylinositol-3-kinase and phospholipase C-γ as possible targets (ten Freyhaus et al., 2015). Further research along these lines are clearly warranted to safely and effectively target the PDGF pathway in PAH.

We further asked whether we can utilize the fact that our 3D-PAH media model allows the measurement of tissue thickness to assess the ability of compounds to suppress medial thickening. We have provided a proof-of-concept for the potential utility of our model for this purpose by investigating the effects of bosentan, MRE-269, and tadalafil on the PDGF-induced thickening of our 3D-PAH tissues. These compounds represent each of the three main signaling pathways targeted in current PAH treatment (Humbert et al., 2004). Our results revealed that all three compounds mitigated PDGF signaling-mediated medial thickening in our 3D-PAH media model. This, furthermore, correlated with reduced expression of proliferation markers and increased induction of apoptosis. To the best of our knowledge, ours is the first analysis regarding the inhibitory effect of these clinical drugs on PDGF-induced medial thickening and PASMC proliferation in a 3D setting. However, our results are consistent with previous papers demonstrating the suppression of PDGF-induced PASMC proliferation by these clinical PAH drugs or related compounds targeting the same molecular pathway in conventional 2D culture (Kunichika et al., 2004; Tantini et al., 2005; Gatfield et al., 2017). This corroborates the relevance of our novel *in vitro* model of medial thickening in PAH and the use of 3D-PAH media tissue thickness as an output measure to assess the efficacy of candidate compounds. The construction and concomitant evaluation of 3D media tissues generated from healthy PASMCs might be useful in identifying possible toxicities and assessing the specificity of drugs to PAH. This is an avenue of research that warrants to be developed in the future.

Previous analyses have revealed differential gene expression profiles depending on whether cells are cultured in 2D or 3D with 3D culture conditions demonstrating an expression profile more similar to that seen in native tissue (Yu et al., 2018). We thus expect that our 3D-PAH medial thickening model can potentially be used in the future to screen novel compounds for their ability to suppress medial thickening for use against PAH. To this end, a thorough analysis of gene expression profiles in 3D-PAH tissues as compared to conventional 2D culture and/or native tissue is warranted.

## Data Availability Statement

The datasets generated for this study are available on request to the corresponding author.

## Ethics Statement

The studies involving human participants were reviewed and approved by the National Hospital Organization Okayama Medical Center (Okayama, Japan). The patients/participants or their next of kin provided their written informed consent to participate in this study.

## Author Contributions

HT, MY, HM, MK, and AO conceived and designed the experiments. CM, HT, YI, and NN performed the experiments. CM, HT, YI, and AO analyzed the data. HT and AO wrote the manuscript. MK and AO supervised the research. All authors read, reviewed, and approved the final manuscript.

## Conflict of Interest

AO received lecture fees from Actelion Pharmaceuticals Japan Ltd., Nippon Shinyaku Co., Ltd., and Pfizer Japan Inc. HM received lecture fees from Actelion Pharmaceuticals Ltd., Bayer AG, GlaxoSmithKline plc, Grupo Ferrer Internacional, S.A., Nippon Shinyaku Co., Ltd., Pfizer Japan Inc., and United Therapeutics Corporation. The remaining authors declare that the research was conducted in the absence of any commercial or financial relationships that could be construed as a potential conflict of interest.
