# Supplementary material for: 3D in vitro Model of Vascular Medial Thickening in Pulmonary Arterial Hypertension
Source: Front Bioeng Biotechnol. 2020 May 20;8:482. doi: 10.3389/fbioe.2020.00482 (PMC7251161; doi:10.3389/fbioe.2020.00482)
Supplement: Supplementary file 1 [file Data_Sheet_1.pdf]

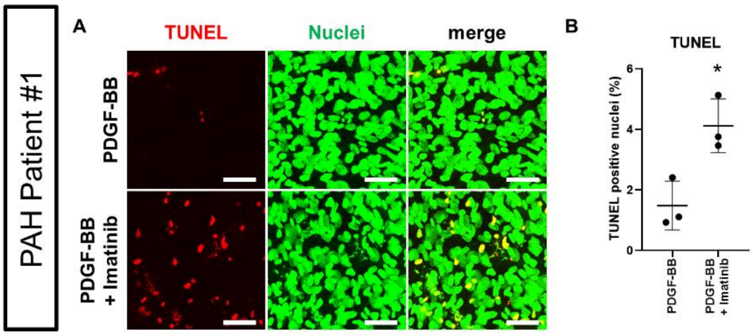

Supplementary Figure 1: Effect of imatinib on PASMC apoptosis in 3D-PAH media tissues. (**A**) Representative terminal deoxynucleotidyl transferase dUTP nick end labeling (TUNEL) staining (red) images of 3D-PAH media tissues cultured in the presence of PDGF-BB with or without imatinib treatment. Nuclei were stained by SYTOX Green (green). Scale bars = 50 µm. (**B**) Quantification of TUNEL-positive nuclear area as a percentage of the total nuclear area as shown in (**A**). *: p<0.05, unpaired Student's *t*-test with Welch's correction. All experiments shown in this figure were performed on PASMCs derived from PAH patient #1.

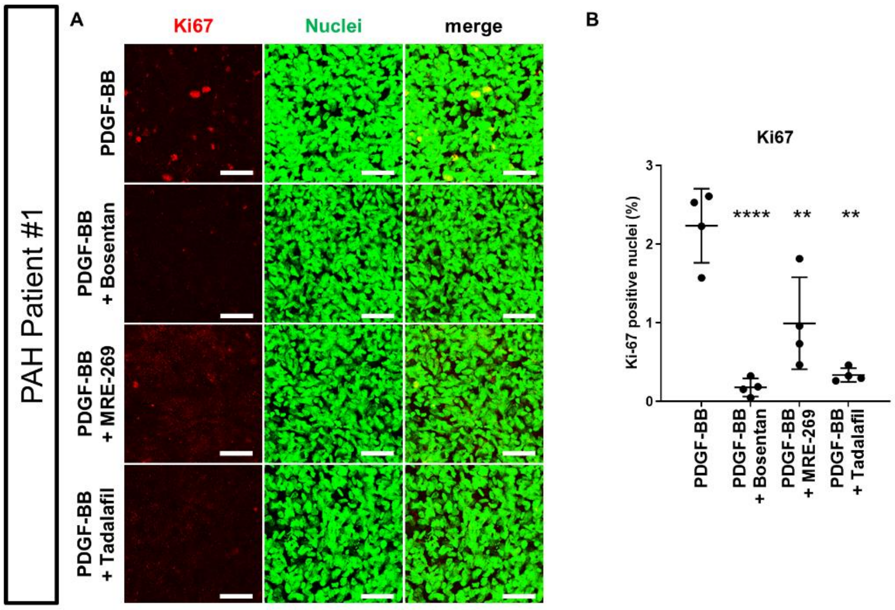

Supplementary Figure 2: Effect of clinical PAH drugs on PASMC proliferation in 3D-PAH media tissues. (**A**) Representative Ki67 staining (red) images of 3D-PAH media tissues cultured in the presence of PDGF-BB with or without bosentan, MRE-269, or tadalafil treatment. Nuclei were stained by SYTOX Green (green). Scale bars = 50 μm. (**B**) Quantification of Ki67-positive nuclear area as a percentage of the total nuclear area as shown in (**A**). **: $p<0.01$ and ****: $p<0.0001$, one-way analysis of variance followed by *post hoc* Dunnett's multiple comparisons test. All experiments shown in this figure were performed on PASMCs derived from PAH patient #1.

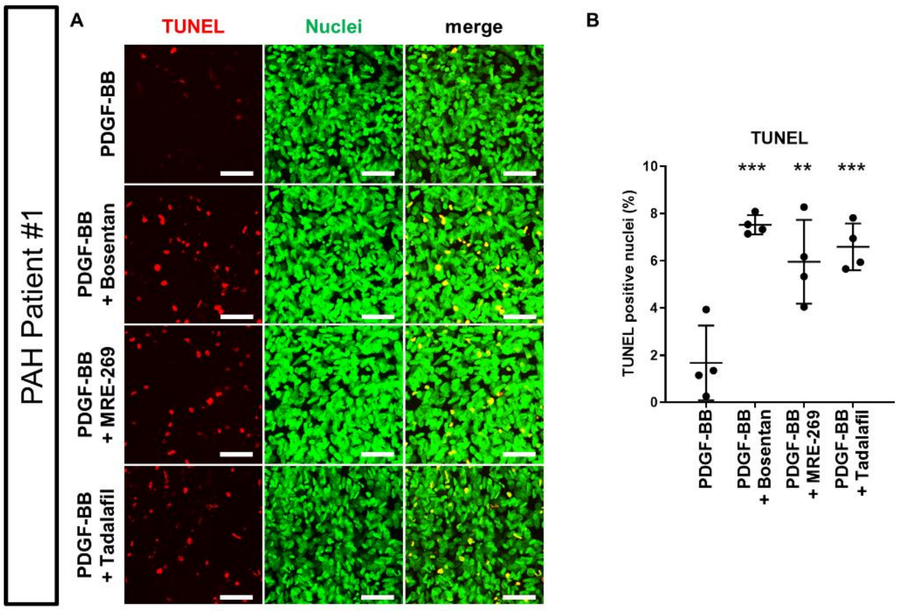

Supplementary Figure 3: Effect of clinical PAH drugs on PASMC apoptosis in 3D-PAH media tissues. (**A**) Representative TUNEL staining (red) images of 3D-PAH media tissues cultured in the presence of PDGF-BB with or without bosentan, MRE-269, or tadalafil treatment. Nuclei were stained by SYTOX Green (green). Scale bars = 50 μm. (**B**) Quantification of TUNEL-positive nuclear area as a percentage of the total nuclear area as shown in (**A**). **: $p<0.01$ and ***: $p<0.001$, one-way analysis of variance followed by *post hoc* Dunnett's multiple comparisons test. All experiments shown in this figure were performed on PASMCs derived from PAH patient #1.
